# Expanding the 3 Wishes Project for compassionate end-of-life care: a qualitative evaluation of local adaptations

**DOI:** 10.1186/s12904-020-00601-5

**Published:** 2020-06-30

**Authors:** Meredith Vanstone, Thanh H. Neville, Marilyn E. Swinton, Marina Sadik, France J. Clarke, Allana LeBlanc, Benjamin Tam, Alyson Takaoka, Neala Hoad, Jennifer Hancock, Sarah McMullen, Brenda Reeve, William Dechert, Orla M. Smith, Gyan Sandhu, Julie Lockington, Deborah J. Cook

**Affiliations:** 1 Department of Family Medicine, McMaster University, Hamilton, Ontario Canada; 2 Department of Medicine, Division of Pulmonary & Critical Care, University of California Los Angeles, California, Los Angeles USA; 3 Department of Health Research Methods, Evidence, and Impact, McMaster University, Hamilton, Ontario Canada; 4 Vancouver Coastal Health, Vancouver, British Columbia Canada; 5 Department of Medicine, McMaster University, Hamilton, Canada; 6 Department of Critical Care, St. Joseph’s Healthcare Hamilton, Hamilton, Ontario Canada; 7 Department of Medicine, Dalhousie University, Halifax, Canada; 8 Department of Medicine, Brantford General Hospital, Brantford, Ontario Canada; 9 Brantford General Hospital, Brantford, Ontario Canada; 10 Critical Care Department, St. Michael’s Hospital, Toronto, Ontario Canada; 11 Departments of Medicine and Health Research Methods, Evidence, and Impact, McMaster University, Hamilton, Ontario Canada

**Keywords:** Intensive care unit, End of life care, Empathy, Program evaluation, Qualitative research

## Abstract

**Background:**

The 3 Wishes Project (3WP) is an end-of-life program that honors the dignity of dying patients by fostering meaningful connections among patients, families, and clinicians. Since 2013, it has become embedded in the culture of end-of-life care in over 20 ICUs across North America. The purpose of the current study is to describe the variation in implementation of 3WP across sites, in order to ascertain which factors facilitated multicenter implementation, which factors remain consistent across sites, and which may be adapted to suit local needs.

**Methods:**

Using the methodology of qualitative description, we collected interview and focus group data from 85 clinicians who participated in the successful initiation and sustainment of 3WP in 9 ICUs. We describe the transition between different models of 3WP implementation, from core clinical program to the incorporation of various research activities. We describe various sources of financial and in-kind resources accessed to support the program.

**Results:**

Beyond sharing a common goal of improving end-of-life care, sites varied considerably in organizational context, staff complement, and resources. Despite these differences, the program was successfully implemented at each site and eventually evolved from a clinical or research intervention to a general approach to end-of-life care. Key to this success was flexibility and the empowerment of frontline staff to tailor the program to address identified needs with available resources. This adaptability was fueled by cross-pollination of ideas within and outside of each site, resulting in the establishment of a network of like-minded individuals with a shared purpose.

**Conclusions:**

The successful initiation and sustainment of 3WP relied on local adaptations to suit organizational needs and resources. The semi-structured nature of the program facilitated these adaptations, encouraged creative and important ways of relating within local clinical cultures, and reinforced the main tenet of the program: meaningful human connection at the end of life. Local adaptations also encouraged a team approach to care, supplementing the typical patient-clinician dyad by explicitly empowering the healthcare team to collectively recognize and respond to the needs of dying patients, families, and each other.

**Trial registration:**

NCT04147169, retrospectively registered with clinicaltrials.gov on October 31, 2019.

## Background

The 3 Wishes Project (3WP) is an end-of-life program designed to attenuate human suffering by cultivating connections and deepening relationships among patients, family members, and clinicians in the intensive care unit (ICU) [1–3]. These relationships are fostered through a focus on the preferences and legacy of the dying patient [3–5]. The 3WP begins with clinicians asking how they might honor the patient in the last moments of life and bring comfort to the family, then evolves to working with other clinicians, family members, volunteers, and the community to implement these wishes. The program began in 2013 at St. Joseph’s Healthcare in Hamilton, Ontario. Today, the 3 Wishes Project has not only become embedded into the culture of end-of-life care in the original unit, but thrives in over 20 ICUs across North America in a variety of formats ranging from research project to clinical program. Through a variety of mixed-methods studies, we have demonstrated the impact of the 3WP as experienced by families, clinicians, and trainees [6–11]. Recently, we published a multicenter formative program evaluation of the 3WP in 4 hospitals, demonstrating it is a transferrable, affordable, and sustainable end-of-life care program that provides value to families, clinicians, and the institution [9].

As 3WP continues to expand, the original 3WP site receives numerous requests for advice about initiation in new centers. Many requests relate to how new sites might implement 3WP with limited resources, and how the program might operate without the research elements and the associated infrastructure of an academic tertiary care hospital. With the goal of assisting new centers considering local implementation of 3WP, the objective of this study is to describe various designs of the 3WP across 9 ICUs in North America, to identify essential elements for success, and to describe local adaptations.

## Methods

We conducted a qualitative descriptive [12] study of the process of implementing this semi-structured end-of-life clinical program in 9 ICUs across 7 hospital systems within Canada and the United States. This research was aligned with a pragmatist epistemological paradigm [13]. A qualitative descriptive approach is appropriate for this type of applied health research question because it allows analysis to stay closely connected to participant words and ideas, without significant interpretive inference.

The expansion of the 3WP was guided by implementation support from the original center in the form of a Start-Up Guide [14], publications [7, 9], periodic teleconferences, email, and/or site visits. As the program spread, the original center encouraged new sites to adapt and refine the program to address their units’ identified needs with available resources, so long as the program continued to be guided by conversations with dying patients (if able) and their families about what would be meaningful at the end of life. These “wishes” were then implemented by the care team, other hospital staff, family members, and friends.

Participating centers were sampled from amongst centers where 3WP was successfully implemented and data was shared back to the original center. We allowed each center to self-define “successful implementation”, because we wanted a sample of centers with different levels of experience with the program, from different sizes of hospitals so imposing a definition of success related to the number of patients enrolled or longevity at the center would not be helpful. We sampled participating centers to represent diversity in the model of 3WP implemented, duration of implementation, and organizational and geographical context. All centers collected data describing patient characteristics and wishes implemented. At some centers, quantitative data were supplemented with qualitative data about the experiences of family members and clinicians. Supplemental Figure A describes different implementation models, including an approach that did not involve data collection (a model not represented in this study, as this report is data-based).

Ethics approval for this study was obtained from the Hamilton Integrated Research Ethics Board and from each institutional review board.

From 2016 to 2019, we conducted interviews and focus groups with individuals active in the initiation or day-to-day leadership of 3WP at each of the 7 hospitals. Focus groups were composed of individuals from multiple centers to facilitate conversations about local variations. Interviews included one or two key personnel from each center to generate an in-depth description of the program at their site. Interview and focus group guides were designed and piloted with members from the original center and further refined as the project progressed. All interviews and focus groups were conducted by trained qualitative researchers who do not have clinical roles (MV and MES) or bedside implementation experience, but who have long standing familiarity with the program. Interviews and focus groups were audio-recorded and transcribed verbatim; all participants provided written informed consent.

Interview and focus group data were supplemented with process documents produced by the original center. After preliminary analysis, we returned to center leads at each hospital, asking for additional input on several key concepts identified during analysis where data were incomplete. Review of findings and provision of supplemental information where necessary served as a form of member checking to ensure resonance and credibility [15]; and also an opportunity for leads to provide updated information about the evolution of the program at their hospital. Data sufficiency was determined by the ability to create a rich description of the program at each hospital, and local affirmation that no key elements were missing.

Data were analyzed using an iterative, step-wise approach adapted from Grounded Theory [16]. Preliminary coding was completed by two independent coders (MS and MES), who met with a larger multidisciplinary team (MV, DJC, FJC, NH) to review key ideas and decide on a coding schema for subsequent rounds of analysis. Focused coding followed this trajectory, with two coders working independently and a larger group reviewing the work and making decisions about ideas to investigate in future rounds. Findings were triangulated among different sources of data (e.g. interviews, focus groups, process documents) [17] and in the member-checking exercise [15]. N-Vivo 12 was used to manage data.

## Results

**Fig. 1:**
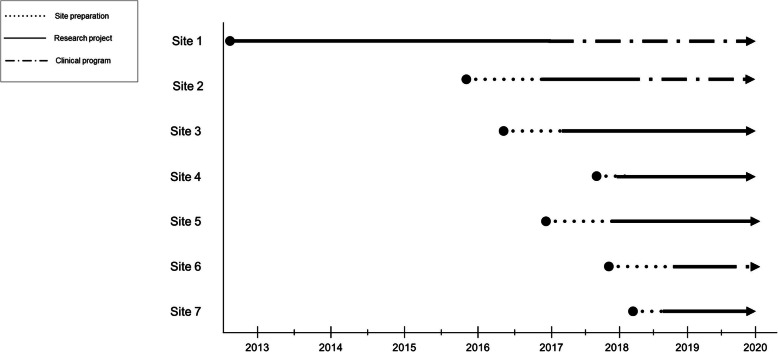
Timeline of Site Participation. In this figure we display the time course associated with initiating the 3 Wishes Project in each of these participating institutions, and the transition from a research project and clinical program to a clinical program only

Figure 1 displays the timeline over which each center implemented the program and collected data. Table 1 describes the features of the 9 ICUs located in 7 hospital systems. Table 2 outlines the roles of the 85 interview and focus group participants, who represented nurses, physicians, spiritual care clinicians, recreational therapists, and others.

**Table 1:** Site Characteristics

Site Characteristics	Center 1 SJH	Center 2 SMH	Center 3 VGH	Center 4 UCLA		Center 5 BGH	Center 6 NH	Center 7 HFX	
Site Sub-Number				**Site 4A** **Ronald Reagan**	**Site 4B** **Santa** **Monica**			**Site 7A** **Victoria** **General**	**Site 7B** **Halifax** **Infirmary**
**Hospital Characteristics**									
Country	Canada	Canada	Canada	USA	USA	Canada	Canada	Canada	Canada
Hospital size (#beds)	570	455	955	520	281	260	495	214	433
Type of Hospital	Academic	Academic	Academic	Academic	Academic	Community	Community	Academic	Academic
Faith-based institution	Catholic	Catholic	Secular	Secular	Secular	Secular	Secular	Secular	Secular
ICU Size (#beds)	23	24 (MSICU)	34	24	20	15	20	8	13
		19 (NTICU)							
Type of ICU	Medical-surgical	Medical-surgical, neuro-trauma	Medical-surgical, neuro-trauma	Medical	Medical-surgical	Medical-surgical	Medical-surgical	Medical-surgical	Medical-surgical, neuro-surgical, trauma
Trainees Present	Yes	Yes	Yes	Yes	Yes	No	Yes	Yes	Yes
**3WP Characteristics**									
Date of initiation	Jan 2013	Oct 2015	April 2016	Dec 2017	Nov 2018	Dec 2016	Oct 2017	Feb 2018	Feb 2018
Background of core 3WP team	1 MD	1 MD	2 MDs	2 MDs	2 MDs	1 SCC	1 MD	2 MD (work in both sites)	
	1 RN	2 RN	1 RN	2 RN	2 RN	1 RT	3 RN	1 Unit Resource RN	1 Unit Resource RN
	1 RC	1 RN Manager	1 CNS	1 CNS	1 RC	1 RN Manager	1 RC	1 SW	1 SCC
	1 SCC	3 RC	1 RN Manager	1 RN Manager		2 MD		1 RN	1 RN
		5 SW	1 RC	1 RC		1 RC			
		2 SCC	1 SCC			1 RN			
			1 SW						
3WP Model	C-2	C-2	C-2	C-2	B	B	B	B	
Prior trainee at SJH	N/A	No	No	No	No	Spiritual Care Resident	Critical Care Medicine Clinical Scholar	Internal Medicine Medical Resident	

In this table, we show characteristics of participating institutions and the 3 Wishes Project teams therein. The 3WP operates in 2 ICUs within one hospital (center 3). The 3WP operates in 2 different sites under the umbrella of one hospital (at both center 4 and center 7). In other words, the pair of Sites 4A and 4B, and the pair of Sites 7A and 7B each represent one health system that has implemented 3WP in two wards in different hospitals. Initiation refers to date of initial engagement with the original site’s 3WP methods center. For explanation of the different 3WP models, please see Supplemental Figure A

(*MD* medical doctor, *RN* registered nurse, *RC* research coordinator, *SW* social worker, *SCC* spiritual care clinician, *CNS* clinical nurse specialist)

**Table 2:** Roles of Individual Interview and Focus Group Participants

Roles	N (%)
Physician	21 (24.7)
Nurse	48 (56.5)
Social Worker	4 (4.7)
Spiritual Care Clinician	4 (4.7)
Clinical Nurse Specialist	4 (4.7)
Research Personnel	3 (3.5)
Recreational Therapist	1 (1.1)
**Total**	**85 Participants**

In this table, we show characteristics of participants in the interviews and focus groups contributing qualitative data to this study

All centers identified a common goal for project initiation: a desire to improve end-of-life care. In addition to identifying essential elements related to this goal, we also describe variations across sites, mainly related to organizational context and resources. Finally, we describe a consistent pattern of program evolution, wherein local adaptations and refinements were facilitated through cross-pollination of ideas fostered by a network of like-minded individuals with shared purpose.

### Essential elements common across sites

We identified several essential elements of 3WP that were present across all centers. The program is founded upon the importance of authentic connections among individuals in the ICU: “*The best part of it is talking with people. It’s not the actual doing of the wish, it’s the conversation with the family that I find the most meaningful*” (RC). These connections are strengthened by active demonstrations of compassion: “*I often talk about [3WP] as crowd-sourcing compassion. … We share a common desire to be compassionate towards patients and families*” (RN). The grassroots, clinician-led nature of the program with wide participation from all members of the clinical team is consistent across centers: “*This project, specifically, has connected us in a way that’s very different from day to day*” (RN). This embeddedness ensures the program is part of usual care, rather than compartmentalized as something “extra”: “*I didn’t even know it was a program, I thought it was ingrained into clinical practice in the ICU.*” (Resident).

### Description of variation across sites

The main points of variation across centers related to the organizational context and resources with which the program was implemented. These differences created diverse foundations upon which local versions of 3WP were built.

#### Organizational context

Differences across participating sites provoked discussion of practical and logistical issues. For example, smaller centers with fewer staff members found it challenging to mobilize individuals to implement wishes due to competing clinical demands: “*We’re so short-staffed, so I don’t know how often those kinds of things occur cause, like if eight people are working that means only three people can go sit down and have a* [3WP] *meeting.”* (MD)*.*

Larger centers also faced communication and mobilization challenges, but for different reasons. While it was easy to identify 3WP champions from a large pool of individuals, communicating about the program to the full staff complement was more difficult. “*Honestly, we’re talking several hundred people that we’re trying to talk to*.” (RN) This site also described needing to do a periodic “*educational blitz*” (RN) to reach new staff and keep awareness of the program at the top of everyone’s mind.

Each center identified different features of their ICU that facilitated the start and spread of the program. For some academic centers, it was a research tradition and the accompanying infrastructure “*Any university that is accustomed to having lots of research studies … it’s easier to start than having to prove the benefits beforehand.*” (MD). For other sites, it was an institutional mission statement that focused on high quality end-of-life care or the importance of a positive patient experience. Others identified particular strengths or resources of team members, such as having “*a very young staff*” (RN) enthusiastic about initiating new projects, or having a culture that emphasized the importance of palliative care “*end-of-life is not something that most of our group shy away from*” (MD).

Level of administrative support varied, with some centers emphasizing the key roles that clinical managers played in creating space and time to initiate 3WP, “*When this came up he* [Manager] *threw his support behind it 150 percent*” (RN), while others emphasized the initial challenge posed by management before they understood the nature and value of the program. Describing an early attempt to implement a patient’s wish of seeing his pet one last time, one clinical leader recalled asking for permission to bring the animal into the ICU. The person he asked “*emailed his boss and then it turned into… ‘you need to get the CEO, every single leader and also the whole [University] to sign off, this is a universal policy’*” (MD). More commonly, the initiation of 3WP was described as outside of the attention of administrators: “*I’m sure they don’t know what 3 Wishes is, I don’t think anyone knows or cares what we’re doing*” (MD). Typically, as the program continued, administrators became more aware of it, and were described as supportive - either passively or actively.

Each center had a core team of clinicians with different backgrounds and of different sizes who started the 3WP: “*Our social workers, our spiritual care providers, our nurses do the asking part whereas the research team would collect the data. The research team often did the running if something required running*” (RC). All sites noted that their program was initiated and run by front-line clinicians, and the team composition evolved over time: “*One of the things that was intentional but looked organic was that it started out as an intensive care, palliative care, spiritual care collaboration with nursing, respiratory therapy, so it was very interprofessional from inception*” (MD). Nursing staff were commonly described as key to the 3WP, especially in smaller centers: “*Because we’re a community ICU, the bulk of our staff that are involved with patients are physicians and nurses just based on who’s available, because our interdisciplinary team is stretched thinner across the hospital than perhaps in the bigger sites, and so they’re not getting to know the patients as intensely as the nurses and physicians are.”* (RN).

#### Resources

All sites were required to creatively identify and secure resources to support the 3WP, but strategies and successes differed. Some resources were readily available: staff enthusiasm, community contributions, and volunteer energy. “*During various times we actually have a lot of* [volunteer] *people there, so they helped us a lot. These are students from the university … we actually utilize them a lot to help us gather supplies or to help us.*” (RN). Other resources had to be creatively sourced. Organizing financial resources required ingenuity, with teams securing funds from research grants, awards, fundraising among staff and the hospital community, and small corporate grants for supplies, as outlined in Table 3. Several sites successfully tapped into resources beyond the ICU, such as musicians in the volunteer association. Others connected with groups such as school children who made blankets to comfort dying patients, forming ongoing relationships that benefitted both parties: “*The [school] board said ‘yup, we love it, it’s a great idea, but we in turn would like someone from the hospital to come and talk about death and dying and why the program is important’*” (MD). Provision of supplies and services from local coffee shops and other stores was facilitated by forming relationships with local businesses. At one site, the hospital provided support from their operating budget: “*a 3 Wishes Project Manager was hired by administration after a little over a year of 3WP implementation when they saw the value of the project.*” (MD).

**Table 3:** Sources of Monetary and In-kind Funding for the 3 Wishes Project

Source of Funding	Center 1 SJH	Center 2 SMH	Center 3 VGH	Center 4 UCLA	Center 5 BGH	Center 6 NH	Center 7 HFX
**Cash**							
***Individual Donations***							
Site-initiated fundraising	X		X	X	X		X
Donations from family and friends of deceased patients	X		X	X	X		X
Philanthropy from staff and community members	X	X	X	X	X	X	
***Organizational Funds***							
Internal hospital grant				X			
Hospital operational funds	X			X			X
Hospital Foundation funds				X	X		X
Hospital Volunteer Association funds	X	X		X		X	
***Grants***							
Peer review grant	X	X	X	X		X	
Corporate community grant	X					X	
Internal research funds	X	X		X	X		X
Cash awards	X	X	X				
**In-Kind**							
Donations from family and friends of deceased patients (e.g., candles)	X		X	X	X		x
Donations by staff (e.g., music, nail polish)	X		X	X	X	X	X
Community members (e.g., blankets)	X		X	X	X	X	X
Hospital Volunteer Association support staff (e.g., musicians)	X	X		X			
Hospital corporate donations (e.g., parking passes)	X	X		X	X	X	
Other corporate donations (e.g., coffee shop)	X		X	X	X	X	

In this table, we show sources of funding (cash and in-kind) from various sources that support the 3 Wishes Project in participating centers

### Evolution of the 3WP

In each center, the program has evolved over time, adapting to changing needs, transitions in the clinician leadership team, and new resources. These changes have been supported by cross-pollination of ideas in a network of like-minded individuals both within and across hospitals.

#### Local adaptations

Each site shared many local adaptations relating to the way they identified program assets, communicated about the program, involved trainees, and interacted with units outside of the ICU. Many centers developed their own “signature wish”, or a popular wish that was frequently offered by the staff to families. Signature wishes such as word clouds, plaster casts of clasped hands, and thumbprint keychains or jewelry were often developed and popularized at one site and then shared with other centers through the 3WP network.

Experienced sites noted different stages as the 3WP evolved: “*There are definitely clear phases. An implementation phase that involved fundraising, networking, problem solving and demonstrating that 3WP was safe, effective, beneficial, and feasible. We are now in a maintenance phase where we provide ongoing reinforcement of existing knowledge and look for ways to grow the program.*” (RN). Between the implementation and maintenance phase exists a time wherein the program transitions “*into part of the unit culture and [is] incorporated into the standard of care for our end-of-life patients*” (RN). Others described expansion as part of the latter phase of the project, with several sites mentioning that they have expanded the clinical program to other units within their hospital, or affiliated institutional sites: “*We have now expanded to 6 other units in a two-hospital health system.*” (MD).

We also identified a shift in thinking about the program, from a defined clinical and/or research project with specific initiation and enrollment criteria to an organic, engrained approach to end-of-life care. Determining the eligibility of a specific patient and increasing staff comfort with introducing the program was a common initial focus: “*People feel awkward to initiate the conversation and to get from the family what’s most important for that person*” (RN). This initial hesitancy dissipated with experience supported by creating local resources (e.g. coaching by unit champions, lists of example wishes): “*We developed these resources that people leaned on very heavily when they first started out*” (MD). As the program became more established, there was less reliance on the core team as “*the skills and capacity increase amongst the staff*” (RN). The final stage occurs when 3WP is framed as an explicit approach to caring through acts of compassion rather than a program. This was marked by a different approach when speaking with families: “*I just say ‘Would it be ok if we provide a blanket for your mom’?*” (RC) “*It’s like a gentle approach. ‘What did they like and what did they not like?’ or ‘Do you have a pet?’ and you start getting the ideas going*” (RN). Instead of inviting a patient to participate in the 3WP, one site opens with the question, “*Is there anything else that we can do for you right now?*” (RN).

#### Cross-pollination of ideas

Each site discussed the importance of sharing stories and coordinating efforts within their own ICU: “*It could just be one story that affects hundreds of providers, so whatever allows you to have these meaningful patient interactions that people see and want to share”* (MD)*.* Storytelling helps to sustain enthusiasm for the program and encourages participation: “*Word of mouth is the most powerful way to communicate*” (Organ Donation Coordinator). This communication is typically direct and one-to-one. Coordination and logistics are shared within the ICU through staff huddles, meetings, email, protocol binders, research newsletters and bulletin boards, core team group meetings, and group chats (e.g. WhatsApp Messenger). Encrypted group text chains are often used to coordinate wishes, offering the opportunity to ask a large group for help when items outside the hospital are needed: “*They* [program champions] *have a really good network and then if sometimes their work gets tied up they may text someone else who is coming in: ‘Can you stop by and pick up flowers or a treat from* [local store]*?’*” (RN).

Communicating about 3WP to others outside of the ICU but within the institution was considered important, often with the intent of both sharing stories and soliciting supportive resources: “*It [the nurse-for-a-day fundraiser] inspired some really good little competition and, and it was just funny. We got other units involved to come make donations, because I mean all our docs usually work at other places … We were trolling down in Emerg to make donations … it was so fun*” (RN). As word of 3WP spread within each center, other units are often interested in implementing elements of the program: “*We have no formal partnerships outside of the ICU but are actively communicating with other units who are interested in starting a similar program*.” (RN). This intra-institutional communication has led to establishing the 3WP in several other wards in multiple centers (e.g. geriatrics, medical step-down, oncology).

Participants from each center reported making connections within their communities outside of the hospital. Sometimes these connections yielded additional resources or assistance in implementing wishes: “*We’ve partnered with a local artisan jeweler and a charity knitting group*” (RN). Many hospitals received local news media coverage about their program which not only disseminated information about the mission of the project, but also inspired volunteer interest, donations, and new project initiation.

Finally, interaction among centers has been an important way to share ideas and solutions to common logistical challenges. Interactions that were initially coordinated by the original center created a multicenter network that continues to share central resources, engage in reverse site-visits, host retreats with program leaders, and communicate through social media. Ideas were further pollinated across centers once they were established; thus, individuals involved in the 3WP no longer relied solely on the original center as a hub, as other sites took the lead in actively developing resources such as instructional staff videos and guides: “*In consultation with social work, spiritual care, nursing, respiratory therapy, and leadership teams we developed the 3W Guide for Staff to clarify wishes that are possible, share visual examples of implemented wishes, provide advice and examples of how to initiate the conversation, elicit wishes, implement wishes, and encourage creativity and engagement amongst staff.*” (RN). New hospitals interested in developing 3WP now connect with a number of existing sites, capitalizing on the overall professional network: “*We’ve had numerous discussions with other ICUs in our area and elsewhere* [lists sites]*. Many of these sites have gone on to implement successful clinical programs.*” (RN).

## Discussion

In this paper we describe the implementation of 3WP in 9 ICUs located in 7 hospital systems, detailing variations in the program across sites, identifying essential elements, and reporting potential adaptations that new centers may find helpful when considering whether or how to implement 3WP. Examining the factors facilitating multicenter implementation reveals the importance of program flexibility and the empowerment of frontline staff. These related elements are both fundamental to successful expansion of 3WP.

Each environment in which the 3WP is implemented is unique, with different organizational features, core team composition, administrative support, academic affiliation, and available resources. The semi-structured nature of the program allows for adaptations that consider the culture and context of each setting [18, 19] and thereby encourages uptake and dissemination [20]. The involvement of frontline clinicians ensures that the program is adapted in a way that would be unlikely with a strictly protocolized intervention implemented in a top-down manner by administrators. The empowerment of frontline clinicians to “own” the program encourages staff to take action to positively influence the culture of their workplace, which may account for our prior finding that 3WP enhances professional satisfaction [7–9]. A strictly protocolized program would likely inhibit individuals from identifying creative ways of relating to each other, potentially reducing the value of human connections realized through the program. Current and previous research about 3WP has found the meaning of these human connections to be one of the most important strengths of the program [6, 7, 9–11, 21].

Another key consequence of frontline clinician ownership of the program is the team approach to end-of-life care. This may supplement the typical patient-physician dyad by explicitly empowering other healthcare staff to recognize and respond to the needs of both the dying patients and their family members. Although an interprofessional approach is generally acknowledged as an important component of high quality end-of-life care in ICU [22, 23], other studies have suggested that this approach is impeded by staff discomfort due to lack of training or uncertainty in their perceived role when caring for dying patients [24]. The open, visible nature of 3WP and its frequent focus in workplace conversations helps to maintain and sustain this team approach, allowing the contributions of all clinicians to be recognized and valued. This democratization of leadership opportunities united clinicians in a common focus on patient and family experiences in end-of-life care.

### Strengths and limitations

This analysis of a palliative care program draws on data from 9 ICUs in 7 hospitals with different populations, institutional contexts, and geographical settings. We collected qualitative data from clinicians occupying diverse roles. Analysis was conducted by investigators with varied clinical and professional backgrounds. The longitudinal involvement of the research team ensured familiarity with the program to better identify unique features in each setting. While not planned a priori, this analysis describes how the 3WP was initiated and eventually integrated organically into practice without a rigid protocol in 9 ICUs with their own culture in Canada and the United States. Family perspectives, though previously obtained about different aspects of this program [6, 7, 9–11], were not sought about program implementation; thus, neither family nor deceased patient perspectives are included here.

## Conclusion

In this report, we describe the adaptations and refinements to the 3 Wishes Project that enabled successful implementation and fostered viability in variable ICU settings despite different contexts, needs, and resources. Commitment to high quality end-of-life care was an essential motivator, inspiring frontline clinicians to initiate the program, creatively resource it, and maintain enthusiasm. As the program became more established, it transitioned from an “add on” to existing end-of-life programs to become “embedded into” the unit’s approach to end-of-life care. This evolution was initially facilitated and later fueled by the alliance of like-minded individuals within each site, among several communities, and across the 3WP network.

## Supplementary information

**Additional file 1: Supplemental Figure A.** 3 Wishes Project Implementation Models. Description: There are multiple ways in which an institution can adopt the 3 Wishes Project. This figure illustrates some implementation models for the clinical program and possible variations for an associated research component. Reproduced from Vanstone et al., 2020.

## Data Availability

The qualitative datasets generated during the current study are not publicly available due to the potentially identifying nature of the complete qualitative transcripts and lack of consent from participants to publicly share this data beyond the research team. Accordingly, the data is not available upon request.

## Abbreviations

3WP: 3 Wishes Project
BGH: Brantford General Hospital (Center 5)
CNS: Clinical Nurse Specialist
HFX: Queen Elizabeth II Health Sciences Centre, Halifax (Center 7)
ICU: Intensive Care Unit
MD: Medical Doctor
NH: Niagara Health (Center 6)
RC: Research Coordinator
RN: Registered Nurse
SCC: Spiritual Care Clinician
SJH: St. Joseph’s Health Care Hamilton (Center 1)
SMH: St. Michael’s Hospital Toronto (Center 2)
UCLA: University of California Los Angeles Health System(Center 4)
VGH: Vancouver General Hospital (Center 3)
