# Supplementary material for: Expanding the 3 Wishes Project for compassionate end-of-life care: a qualitative evaluation of local adaptations
Source: BMC Palliat Care. 2020 Jun 30;19:93. doi: 10.1186/s12904-020-00601-5 (PMC7325646; doi:10.1186/s12904-020-00601-5)
Supplement: Supplementary file 1 — Additional file 1: Supplemental Figure A. 3 Wishes Project Implementation Models. Description: There are multiple ways in which an institution can adopt the 3 Wishes Project. This figure illustrates some implementation models for the clinical program and possible variations for an associated research component. Reproduced from Vanstone et al., 2020. [file 12904_2020_601_MOESM1_ESM.docx]

SUPPLEMENTAL FIGURE A: 3 Wishes Project Implementation Models


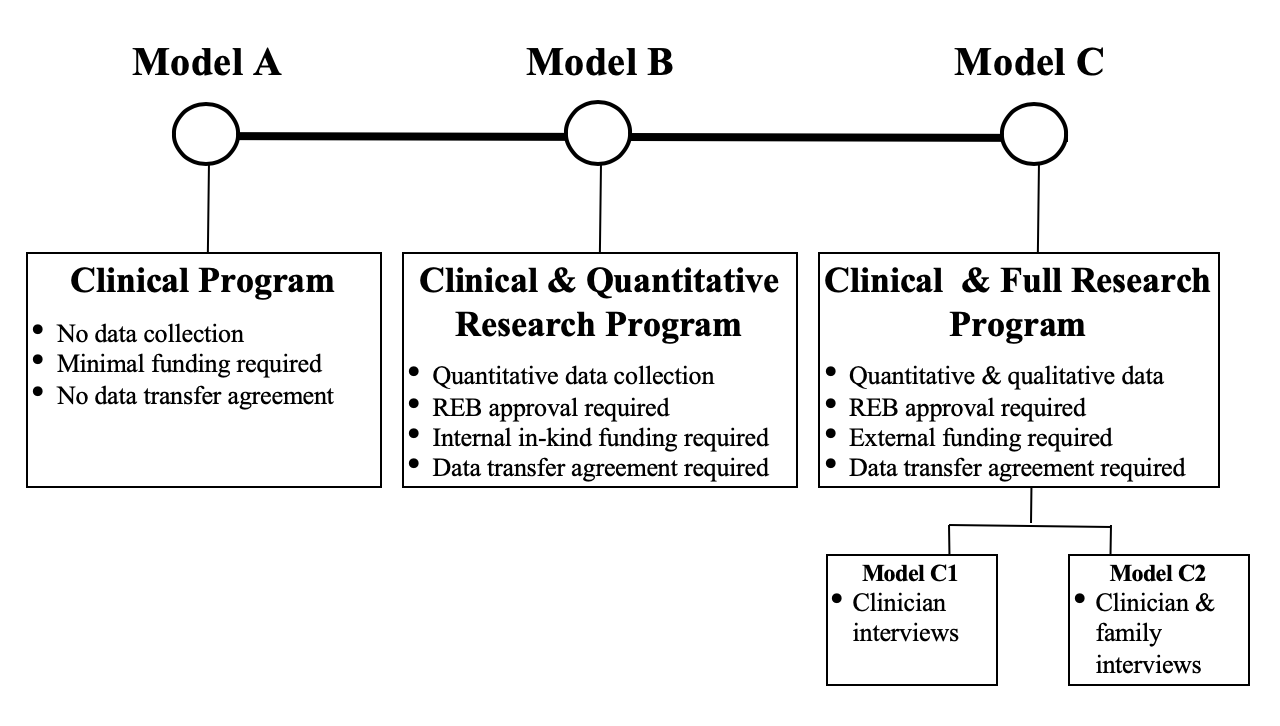


Supplemental Figure A Legend: There are multiple ways in which an institution can adopt the 3 Wishes Project. This figure illustrates some implementation models for the clinical program and possible variations for an associated research component. Reproduced from Vanstone et al, 2020.
